# Overcoming phage resistance: efficacy of sequential phage-colistin therapy against carbapenem-resistant *Acinetobacter baumannii*

**DOI:** 10.1128/spectrum.00855-25

**Published:** 2025-08-14

**Authors:** Yoon-Jung Choi, Md Shamsuzzaman, Chaewon Park, Shukho Kim, Minsang Shin, Jungmin Kim

**Affiliations:** 1 Untreatable Infectious Disease Institute, Kyungpook National University 34986, Daegu, Republic of Korea; 2 Department of Microbiology, School of Medicine, Kyungpook National University 34986, Daegu, Republic of Korea; 3 Department of Biomedical Sciences, The Graduate School, Kyungpook National University 34986, Daegu, Republic of Korea; CEB-Centre of Biological Engineering, Universidade do Minho, Braga, Portugal

**Keywords:** carbapenem-resistant *Acinetobacter baumannii *(CRAB), phage therapy, phage resistance, phage-antibiotic synergy, vB_AbaSt_W16, sequential therapy

## Abstract

Carbapenem-resistant *Acinetobacter baumannii* (CRAB) is a major public health threat due to high mortality and limited treatment options. Bacteriophage (phage) therapy offers a promising alternative, but its long-term efficacy is challenged by the rapid emergence of phage-resistant bacterial populations. This study evaluates phage vB_AbaSt_W16 and whether sequential phage-antibiotic therapy enhances bacterial clearance and suppresses resistance. The optimal multiplicity of infection (MOI) for vB_AbaSt_W16 was determined across various CRAB strains. PAS was assessed *in vitro* using meropenem, colistin, tigecycline, rifampin, and ampicillin/sulbactam. An immunocompromised BALB/c mouse model was used to evaluate the therapeutic efficacy of phage monotherapy and combination therapy. Antibiotics were administered either concurrently with phage or sequentially 6 h after phage treatment. Bacterial clearance, survival rates, and the emergence of phage-resistant populations were monitored. Phage monotherapy inhibited bacterial growth at MOIs of 0.1–10, but high-dose treatment (MOI > 100) caused rapid regrowth within 12 h due to resistance emergence. Phage-antibiotic synergy (PAS) was observed only with tigecycline (FICI = 0.225), though the effect was transient. In the mouse model, phage monotherapy at MOIs of 0.1–1 achieved 100% survival, whereas MOI 10 reduced survival to 40%, correlating with increased bacterial burden and resistance emergence. Notably, sequential administration of colistin 6 h post-phage treatment improved survival to 100%, while colistin monotherapy resulted in survival below 40%. Sequential colistin treatment also effectively suppressed resistant bacterial populations, reducing them to nearly undetectable levels by day 4. High-dose phage treatment was limited by rapid resistance development. In contrast, sequential phage-antibiotic therapy, particularly when combined with colistin, significantly improved bacterial clearance, enhanced survival outcomes, and suppressed resistance emergence. These findings support sequential phage-antibiotic therapy as a strategy for CRAB infections.

**IMPORTANCE:**

The rise of carbapenem-resistant *Acinetobacter baumannii* (CRAB) presents a severe challenge in healthcare settings, where treatment options are increasingly limited. While bacteriophage therapy offers a novel antimicrobial approach, its effectiveness is often hindered by the rapid emergence of phage-resistant bacterial populations. This study demonstrates that sequential phage-antibiotic therapy, particularly phage followed by colistin, significantly improves survival rates and suppresses resistance emergence in a murine infection model. Unlike monotherapies, this strategy optimally combines the bacterial-killing mechanisms of phages and antibiotics, offering a clinically viable solution to combat multidrug-resistant infections. Our findings provide valuable translational insights, supporting the potential application of phage therapy in human medicine. By addressing the critical issue of phage resistance, this study advances the development of sustainable bacteriophage-antibiotic treatment regimens against CRAB and other drug-resistant pathogens.

## INTRODUCTION

The increasing prevalence of multidrug-resistant bacterial infections (MDR) presents a significant challenge to public health, particularly in hospital settings where *Acinetobacter baumannii* thrives (1–3). It causes various nosocomial infections, such as ventilator-associated pneumonia, bloodstream infections, and wound infections, especially in immunocompromised patients. Carbapenem-resistant *A. baumannii* (CRAB), a highly resistant variant of *A. baumannii*, has been classified as a critical priority pathogen by the World Health Organization (WHO) due to its resistance to nearly all available antibiotics, leading to high morbidity and mortality rates (4–8). This growing resistance crisis underscores the urgent need for alternative therapeutic strategies, such as bacteriophage (phage) therapy (9–11). Phages are highly specific, self-replicating agents that bypass conventional resistance mechanisms. Moreover, phage therapy has been shown to work synergistically with antibiotics, potentially restoring the efficacy of certain antimicrobials against resistant bacteria. A key barrier to phage therapy is the rapid emergence of phage-resistant bacteria, which limits clinical application. Bacteria can evade phage infection through receptor mutations, CRISPR-Cas systems, and other adaptive resistance mechanisms, which can significantly reduce the therapeutic efficacy of phage monotherapy (12, 13). To counteract these limitations, combination strategies involving phages and antibiotics have been proposed to enhance bacterial clearance and suppress the development of resistance (14–16). Recent studies have explored phage-antibiotic synergy (PAS) as a strategy to improve treatment outcomes. Certain antibiotics can enhance phage efficacy by increasing bacterial susceptibility, weakening biofilm structures, or inhibiting resistance mechanisms. Additionally, the sequential administration of phages and antibiotics has shown potential in delaying resistance development (17). Although concurrent phage-antibiotic therapy has been explored, few studies have investigated the timing of antibiotic administration following phage treatment. This study specifically assessed a sequential strategy in which antibiotics were administered 6 h after phage exposure before the emergence of resistant populations.

This study focuses on *Acinetobacter* phage vB_AbaSt_W16, a lytic phage with strong activity against CRAB (18). Previous studies have demonstrated that this phage exhibits a high burst size and a short latent period, making it a promising candidate for therapeutic applications. The primary objective of this study is to evaluate the pharmacodynamics of vB_AbaSt_W16 in combination with antibiotics and determine whether sequential phage-antibiotic therapy can enhance bacterial clearance and suppress the emergence of phage-resistant mutants. This study aims to identify treatment schedules that improve efficacy and limit resistance. These findings will provide valuable insights into the clinical feasibility of phage-antibiotic combination therapies for combating MDR *A. baumannii* infections.

## MATERIALS AND METHODS

### Ethical clearance and animal model

This study was conducted in accordance with the Korean Animal Protection Act (Act No. 4379, amended Act No. 19234) and approved by the Institutional Animal Care and Use Committee (IACUC) of Kyungpook National University (Approval No. 2024-0592). Six-week-old female BALB/c mice were maintained under specific pathogen-free conditions with controlled temperature (22 ± 2℃) and humidity (50 ± 10%). Mice were provided an antibiotic-free diet and water *ad libitum*. Anesthesia was administered using 2–2.5% isoflurane, and euthanasia was performed via CO_2_ inhalation following the 3Rs principle (replacement, reduction, refinement).

### Bacterial strains and phage preparation

We used one standard strain of *A. baumannii* (ATCC17978) and four clinical isolates of CRAB, KBN10P02782 (ST552), KBN10P04598 (ST229), LIS20130976 (ST357), and KBN10P04697 (ST784), which were obtained from the Pathogen Resource Bank at Kyungpook National University Hospital. The bacterial strains were cultivated on blood agar plates (BAP, Yeongin, Korea) and in brain heart infusion (BHI) broth and agar (Difco, USA).

Phage vB_AbaSt_W16 was propagated using *A. baumannii* KBN10P02782 and purified using the Phage on Tap (PoT) method (19) with Amicon Ultra-15 centrifugal filters (10 kDa MWCO, Merck Millipore, Ireland). Phage stocks were maintained in SM buffer (50 mM Tris-HCl, 150 mM NaCl, 10 mM MgCl_2_, 2 mM CaCl_2_, pH 7.5) and stored at −25℃.

### Effect of phage vB_AbaSt_W16 on CRAB growth

Bacterial inhibition was assessed using a modified Clinical & Laboratory Standards Institute and Laboratory Standards Institute (CLSI) M07-A10 microdilution assay, a standardized method for antimicrobial susceptibility testing (20). Bacterial cultures were adjusted to an optical density at 600 nm (OD₆₀₀) of 0.05 in Mueller-Hinton broth (MHB) and treated with phage at multiplicities of infection (MOI) ranging from 0.001 to 1,000. Growth was monitored every 6 h over 72 h using a microplate reader (Molecular Devices, USA). Each condition was tested in triplicate, with control wells, including untreated bacterial cultures (positive control) and media-only wells (negative control), to account for background absorbance.

### *In vivo* phage therapy and resistance analysis

Six-week-old female BALB/c mice (16–18 g) were purchased from Orient Bio (Korea) and maintained under specific-pathogen-free conditions with *ad libitum* access to food and water. Neutropenia was induced by intraperitoneal injections of cyclophosphamide (150 mg/kg) on days 3 and 1; body weight and neutrophil counts were verified before infection. Mice were infected intraperitoneally with 10⁸ CFU of *A. baumannii* KBN10P02782 (Fig. S1). After 30 min, groups received intraperitoneal phage treatment at MOIs of 0.01, 0.1, 1, or 10. PBS served as the control. Survival was monitored daily for 7 days to evaluate therapeutic efficacy. Bacterial burden and phage titers in organs (kidney, liver, lung, spleen) were assessed on days 2 and 4.

### Phage-antibiotic synergy (PAS) assessment

The synergistic effects of phage and various antibiotics (meropenem, colistin, ampicillin/sulbactam, tigecycline, and rifampicin) were assessed following modified U.S. CLSI guidelines. Antibiotic concentration ranges included meropenem (0.25–512 µg/mL), colistin, tigecycline, rifampicin (0.25–256 µg/mL), and ampicillin/sulbactam (0.25/0.125–512/256 µg/mL). The synergy test followed the bacteriophage-mediated bacterial growth inhibition assay protocol, with the inclusion of antibiotics. Absorbance (OD_600_) was measured at designated time points, and percentage reduction in bacterial growth was calculated using the formula:


$$Reduction\;(\%)=[(OD_{growth\;control}-OD_{treatment})÷OD_{growth\;control}]\times 100$$

These measurements were averaged after three repetitions to generate synograms. To quantify synergy, the fractional inhibitory concentration (FIC) was calculated:


FIC=MIC of the drug in combination ÷ MIC of the drug alone.

The fractional inhibitory concentration index (FICI) was calculated as follows as the sum of the FIC values of both the antibiotic and the phage:


FICI=FIC of antibiotic+FIC of phage

Synergy was defined as FICI *≤* 0.50, additive effects as 0.50–1.00, indifference as 1.00–2.00, and antagonism as 2.00.

### Enumeration of phage, bacteria, and resistant phage in various organs

Phage titers, viable bacterial counts, and resistant phage populations were measured in four organs (kidney, liver, lung, spleen) on days 2 and 4 post-infection. After euthanasia, the organs were harvested, placed in 1.5 mL Eppendorf tubes, mixed with PBS (3× organ wt), and homogenized using five glass beads. Bacterial and resistant phage samples were diluted in PBS, while phage titration samples were diluted in SM buffer.

Phage enumeration was conducted as follows: homogenized tissue samples were centrifuged at 3,220 ×*g* (Centrifuge 5810 R, Eppendorf, Hamburg, Germany) for 10 min at 4℃, filtered through a 0.22 µm pore-size Whatman syringe filter (Sigma-Aldrich, Missouri, United States), and sterilized with 10% chloroform (21). The filtrate was serially diluted up to 10^−8^ and plated using a modified double-layer agar (DLA) assay for plaque enumeration. Samples were dispensed in 10 µL aliquots and incubated at 37℃ for 24 h for plaque enumeration (22).

Bacterial enumeration was performed using trimethoprim (5 µg/mL) BHA agar plates. Diluted samples were plated and incubated at 37℃ for 24 h before colony countin(22). Phage resistance was evaluated by counting colonies that grew on trimethoprim (5 µg/mL) BHA plates in the presence of phage. Before inoculation, a 0.75% BHI soft agar layer containing 10^10^ PFU/mL of phage was prepared. Resistant colonies were counted after 48 h of incubation, and resistance was calculated as the ratio of resistant colonies to total colonies.

### Phage-antibiotic sequential therapy in mice

Neutropenic mice were infected intraperitoneally with *A. baumannii* KBN10P02780 (1 × 10⁸ CFU/mouse) and divided into five groups: control (SM buffer), phage alone (MOI 10), antibiotic alone (meropenem, 62 µg/mouse or colistin, 2 µg/mouse), simultaneous phage-antibiotic therapy, and sequential therapy. In all treated groups, phage was administered intraperitoneally 30 min after infection. For sequential therapy, antibiotics were administered intraperitoneally 6 h after phage administration, whereas in the simultaneous group, phage and antibiotics were administered concurrently. Comparisons of therapeutic efficacy were made between the sequential therapy group and the corresponding antibiotic monotherapy group to evaluate potential synergistic effects. All treatments were given as a single dose diluted in 200 µL of sterile PBS. Mice were monitored for 7 days, and survival rates were recorded.

### Statistical analysis

Data were analyzed using GraphPad Prism 10.4.1 (GraphPad Software, San Diego, CA, USA). Survival curves were evaluated using Kaplan-Meier analysis, and bacterial/phage counts were compared using two-way analysis of variance (ANOVA). Statistical significance was set at *P* < 0.05.

## RESULTS

### Effect of phage vB_AbaSt_W16 on CRAB growth at different MOIs

**Fig 1:**
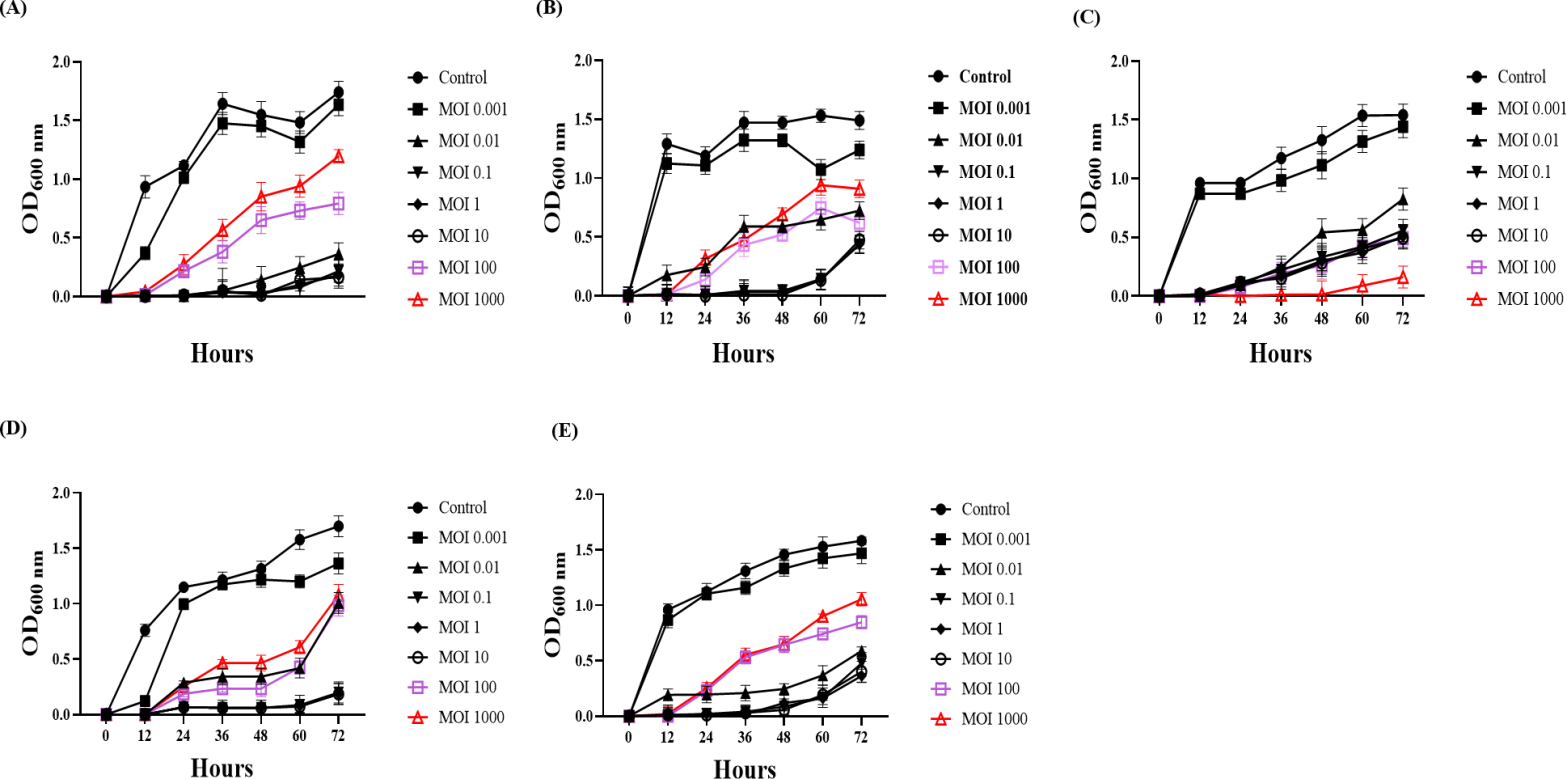
Growth dynamics of strains treated with phage vB_AbaSt_W16 at varying multiplicities of infection (MOI). Bacterial growth was monitored over 72 h by measuring the optical density at 600 nm (OD_600_). (**A**) *A. baumannii* KBN10P02782 (ST552), (**B**) ATCC 17978, (**C**) KBN10P04598 (ST229), (**D**) LIS20130976 (ST357), and (**E**) KBN10P04697 (ST784). MOIs ranged from 0.001 to 1,000 for each strain. Error bars indicate standard deviations from three independent experiments.

To assess the growth-inhibitory activity of phage vB_AbaSt_W16**,** we evaluated CRAB growth across multiple MOIs using OD₆₀₀ measurements over 72 h (Fig. 1). These *in vitro* assays were not intended to simulate therapeutic conditions but to observe inhibition trends. Control groups exhibited typical exponential growth, reaching OD₆₀₀ values of 1.5–2.0 by 72 h. In contrast, phage-treated groups showed dose-dependent suppression, with OD₆₀₀ values remaining below 0.5 at MOIs of 0.01–10 and regrowth observed at MOIs 100 and 1,000 after 12 h. Strain LIS20130976 (Fig. 1C) showed delayed regrowth. While OD₆₀₀ readings above 1.0 may be non-linear due to light scattering, overall trends were consistent. Based on these results and *in vivo* validation, MOIs of 0.01–10 were selected as effective ranges for subsequent animal experiments.

### *In vivo* phage monotherapy and resistance analysis

**Fig 2:**
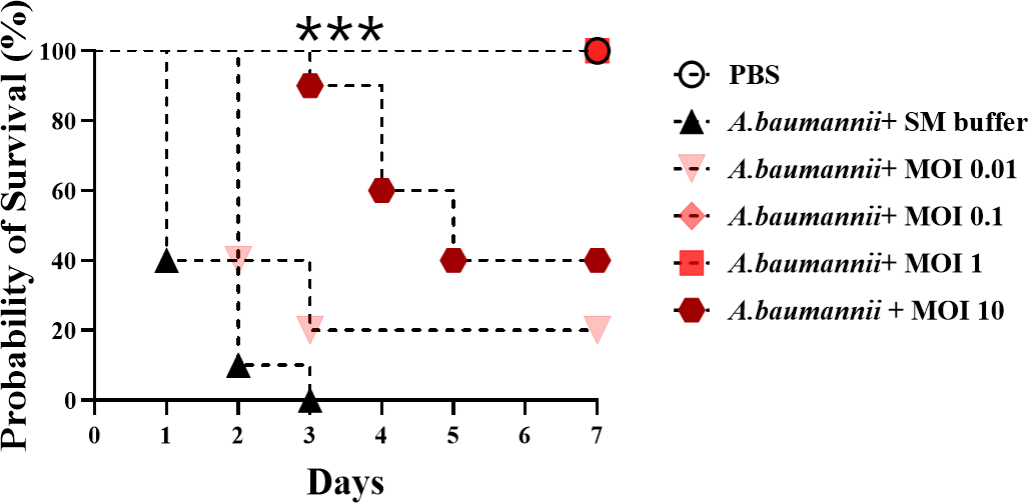
Therapeutic efficacy of phage vB_AbaSt_W16 against systemic infection caused by *A. baumannii* strain KBN10P02782 in neutropenic mice. Neutropenia was induced by intraperitoneal injection of cyclophosphamide (3 mg/mouse) on days 3 and 1. Mice were infected intraperitoneally with *A. baumannii* KBN10P02782 (10^8^ CFU/mouse) and treated 30 min post-infection with phage vB_AbaSt_W16 at MOIs of 0.01, 0.1, 1, or 10. Kaplan-Meier survival curves illustrate survival over a 7-day period (*n* = 15 per group). Statistical analysis was performed using the log-rank (Mantel-Cox) test. Significant differences are indicated by asterisks (***P < 0.001).

The therapeutic efficacy of phage vB_AbaSt_W16 was evaluated in neutropenic and immunocompetent mouse models. High-dose phage (10¹^4^ PFU/mouse) was administered to healthy and neutropenic mice without inducing abnormal signs (Fig. S2). To establish the lethal bacterial dose, neutropenic mice were injected with *A. baumannii* KBN10P02780 at varying concentrations, with 10⁸ CFU/mouse resulting in 100% mortality within 3 days. Based on this, 10⁸ CFU/mouse was chosen for the infection model. Phage was administered 30 min post-infection at MOIs ranging from 0.01 to 10. Although an infected-only control group was initially included in the study design, more than 90% of the mice in this group succumbed to infection before day 2 (as shown in Fig. 2). Therefore, organ-specific bacterial burden and resistance analyses were conducted only in phage-treated groups. By day 7, survival rates were 20% at MOI 0.01, while MOI 0.1 and MOI 1 achieved 100% survival. However, MOI 10 led to a decline in survival, reaching 40% by day 7 (Fig. 2).

**Fig 3:**
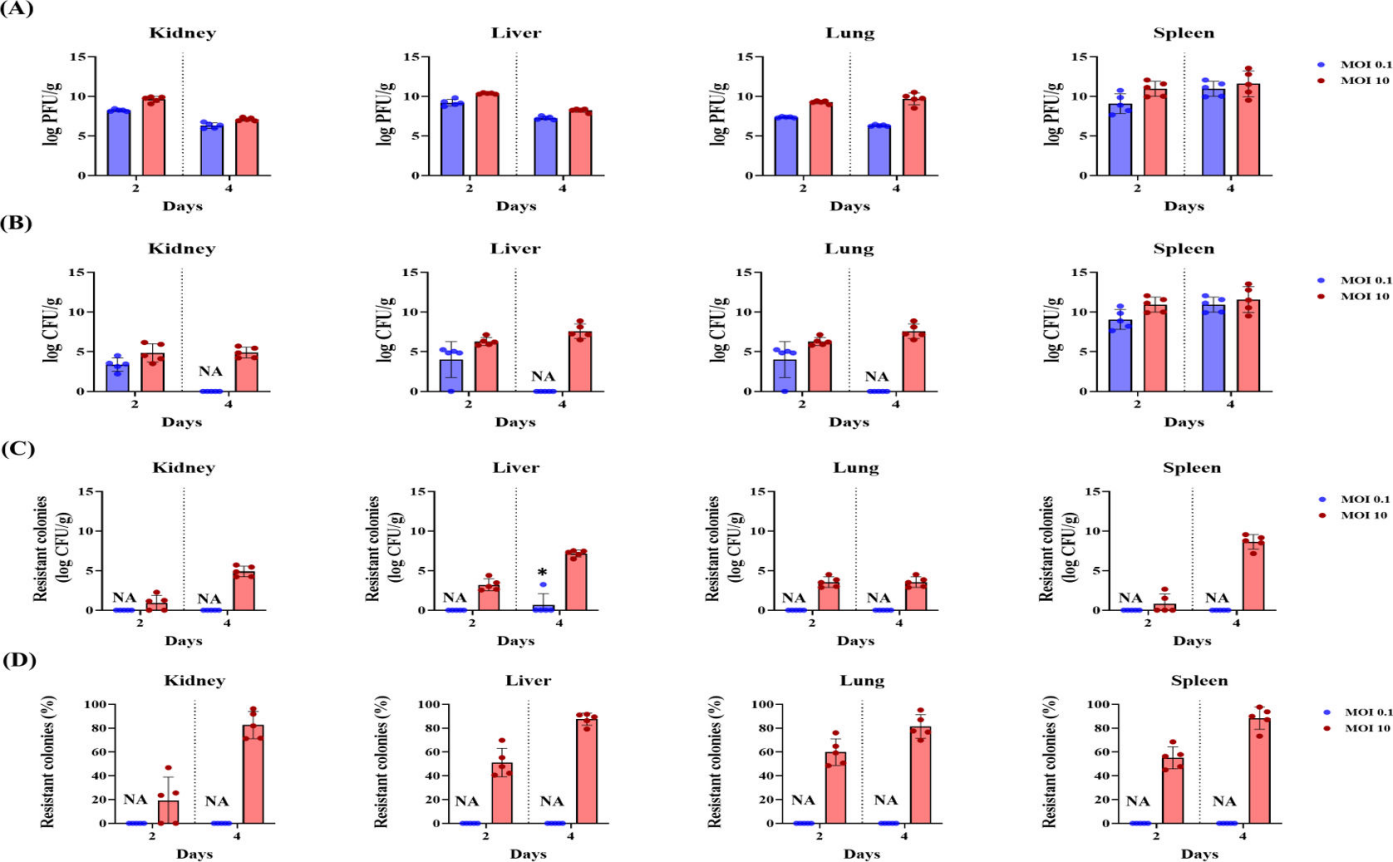
Quantification of phage vB_AbaSt_W16 titers, bacterial burden, and phage-resistant populations in neutropenic mice infected with *A. baumannii* strain KBN10P02782. Mice were treated with phage at MOIs of 0.1 (blue) and 10 (red), and organs (kidneys, liver, lungs, and spleen) were collected on days 2 and 4 post-infection. (**A**) Phage titers (log PFU/g) and (**B**) total bacterial burden (log CFU/g). The infected-only control group was not included in the analysis, as most animals in this group succumbed to infection before day 2. (**C**) Resistant bacterial counts (log CFU/g) and (**D**) resistance ratio (%) calculated as (resistant CFU / total CFU) × 100. When total CFU = 0, the resistance ratio was marked as “NA” (not applicable) and excluded from the statistical analysis. Data represent the mean ± SD of three independent experiments (*n* = 5 per group). Statistical significance was determined using two-way ANOVA, followed by Bonferroni’s post-hoc test. Asterisks indicate significant differences between the MOI 0.1 and MOI 10 groups (*P < 0.05, **P < 0.01).

Phage distribution, bacterial clearance, and resistance emergence were assessed in major organs (kidney, liver, lung, spleen) on days 2 and 4 post-infection (Fig. 3). MOI 10 exhibited 10–100 times higher phage concentrations than MOI 0.1 (Fig. 3A). Resistant colonies began emerging in the MOI 10 group by day 2 and increased significantly by day 4, with CFU counts ranging from 10^2^ to 10^8^ per gram across all organs (Fig. 3C). By day 4, resistant colonies comprised over 90% of the bacterial population in the MOI 10 group while remaining below 80% in the MOI 0.1 group (Fig. 3D).

### Evaluation of phage-antibiotic synergy (PAS)

**Fig 4:**
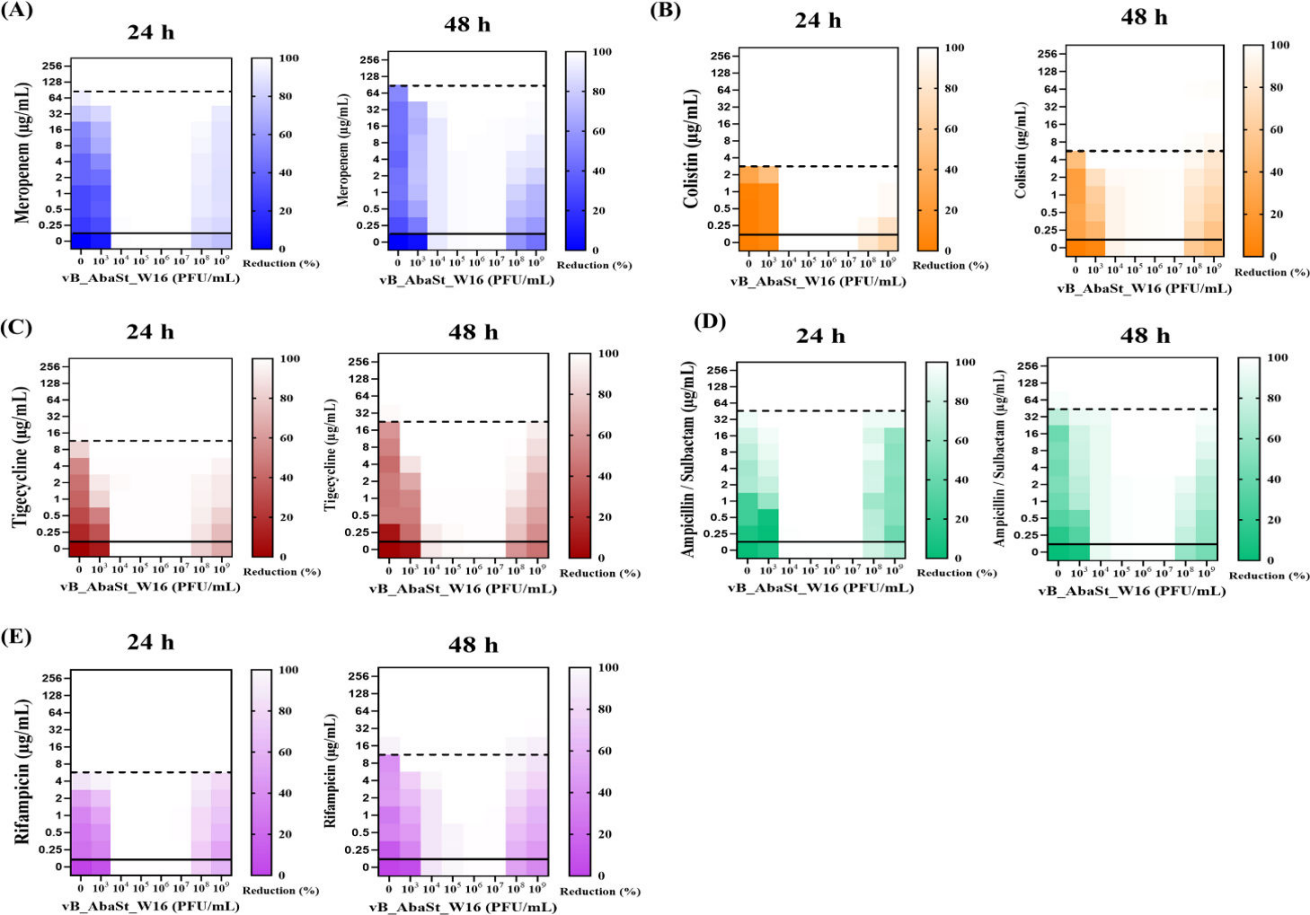
Heatmap analysis of bacterial reduction following phage vB_AbaSt_W16 treatment at varying MOIs in *A. baumannii* LIS20133395 (ST552). Bacterial reduction was measured at 24 and 48 h post-infection in combination with different antibiotics. Panels show suppression profiles for (**A**) meropenem, (**B**) colistin, (**C**) ampicillin/sulbactam, (**D**) tigecycline, and (**E**) rifampicin. Color intensity indicates percentage reduction in bacterial growth; dashed lines represent MIC thresholds. Additional time points (12, 36, 60, and 72 h) are presented in Fig. S2.

The synergy of phage vB_AbaSt_W16 with various antibiotics was analyzed using MIC and FICI assessments at 24 h (Table 1; Fig. 4). The MIC analysis revealed no reduction in MIC for meropenem, colistin, ampicillin/sulbactam, or rifampicin when combined with phage, with FICI values between 1.0 and 2.0 indicating an indifferent effect. However, tigecycline demonstrated a decrease in MIC from 16 to 1 µg/mL, with a FICI of 0.225 signifying synergy. Although tigecycline showed initial MIC reduction, bacterial regrowth occurred after 24 h. Other antibiotics did not exhibit significant bacterial reduction at any time point (Fig. 4).

**TABLE 1:** Antimicrobial susceptibility of *A. baumannii* in the presence of phage vB_AbaSt_W16^*a*^

Antimicrobial against	Antimicrobial susceptibility with phage							
	Antimicrobial susceptibility			Phage susceptibility			FICI	Effects of PAS
	Independent MIC (µg/mL)	Combined MIC (µg/mL)	FIC	Independent MIC (MOI)	Combined MIC (MOI)	FIC		
**Meropenem**	128	128	1.000	0.010	0.001	0.100	1.100	Indifferent
**Colistin**	4	4	1.000	0.010	0.010	1.000	2.000	Indifferent
**Ampicillin/sulbactam**	64/32	64/32	1.000	0.010	0.010	1.000	2.000	Indifferent
**Tigecycline**	16	1	0.125	0.010	0.001	0.100	0.225	Synergy
**Rifampicin**	8	8	1.000	0.010	0.010	1.000	2.000	Indifferent

^*a*^ MIC: minimum inhibitory concentration; FIC: fractional inhibitory concentration; MOI: multiplicity of infection; FICI: fractional inhibitory concentration index; PAS: phage-antibiotic synergy. FIC values below 0.5 are considered ‘synergistic,’ while those above 0.5 are labeled ‘indifferent’.

The synergy between phage vB_AbaSt_W16 and various antibiotics was evaluated using MIC and FICI assessments at 24 h (Table 1; Fig. 4; Fig. S2). At MOI 1.0, phage combinations with meropenem, colistin, ampicillin/sulbactam, or rifampicin showed no significant reduction in MIC values, and the corresponding FICI scores ranged from 1.0 to 2.0, indicating indifferent interactions. In contrast, the phage-tigecycline combination resulted in a marked MIC reduction from 16 to 1 µg/mL with a FICI of 0.225, demonstrating a synergistic activity. However, bacterial regrowth was observed beyond 24 h, suggesting that the synergistic effect was not sustained. Other antibiotic combinations did not produce significant bacterial suppression at any tested time point (Fig. 4; Fig. S2).

### Efficacy of phage-antibiotic sequential therapy against CRAB infection

**Fig 5:**
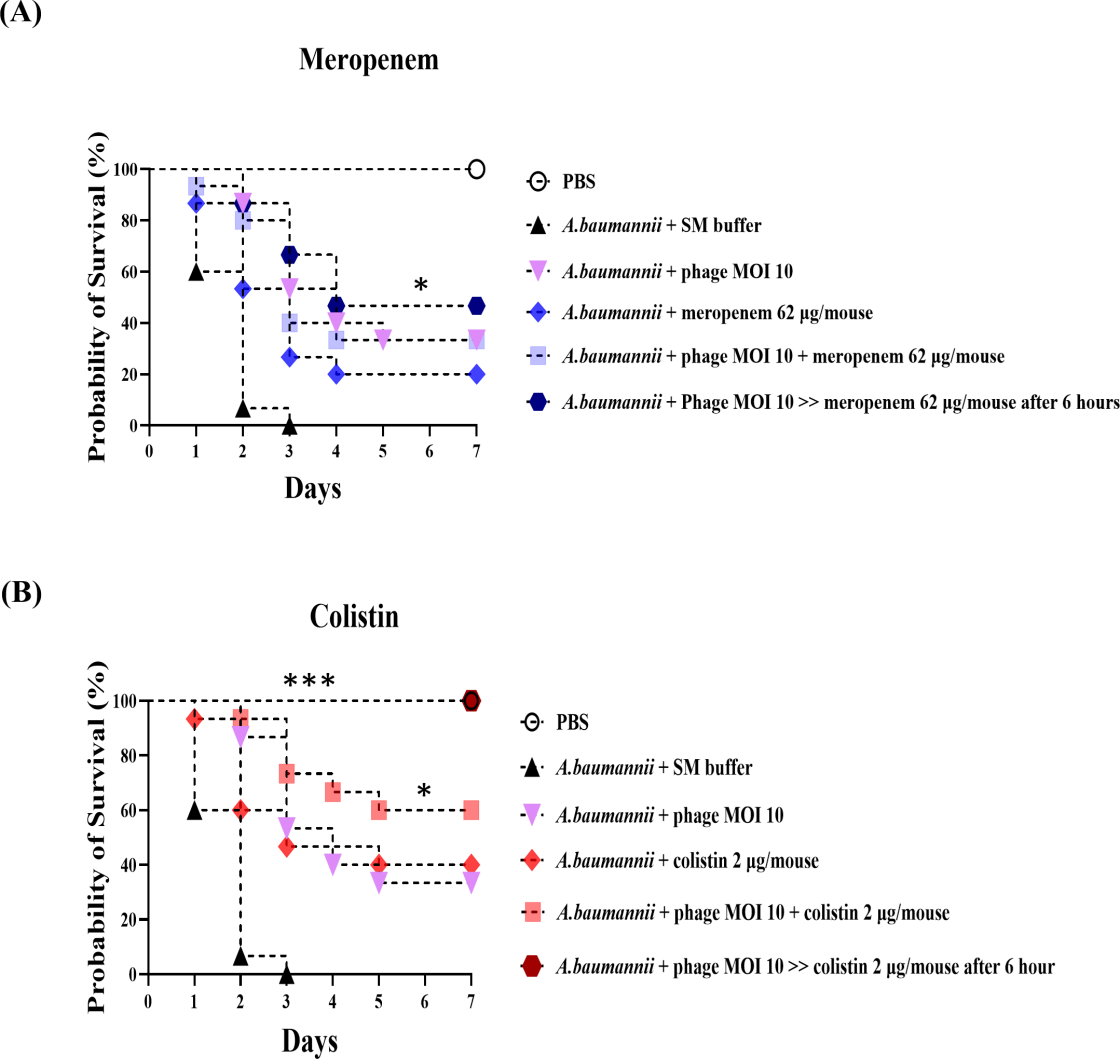
Survival analysis of neutropenic mice infected intraperitoneally with *A. baumannii* strain KBN10P02782 (1 × 10⁸ CFU/mouse). Mice were assigned to five groups: control (SM buffer), phage alone (MOI 10), antibiotic alone (meropenem, 62 µg/mouse or colistin, 2 µg/mouse), simultaneous phage-antibiotic administration, and sequential therapy (antibiotic administered intraperitoneally 6 h after phage). Phage was administered intraperitoneally 30 min after infection in all groups. Treatments were given as a single dose in 200 µL sterile PBS. Survival was monitored over 7 days (*n* = 15 mice/group). Statistical comparisons were made between the sequential therapy group and the corresponding monotherapy group. (**A**) Meropenem groups showed no significant improvement. (**B**) Colistin groups exhibited significantly enhanced survival with sequential therapy. Statistical significance was determined using the log-rank (Mantel-Cox) test. Asterisks indicate levels of significance: **P < 0.05 and ***P < 0.001.

**Fig 6:**
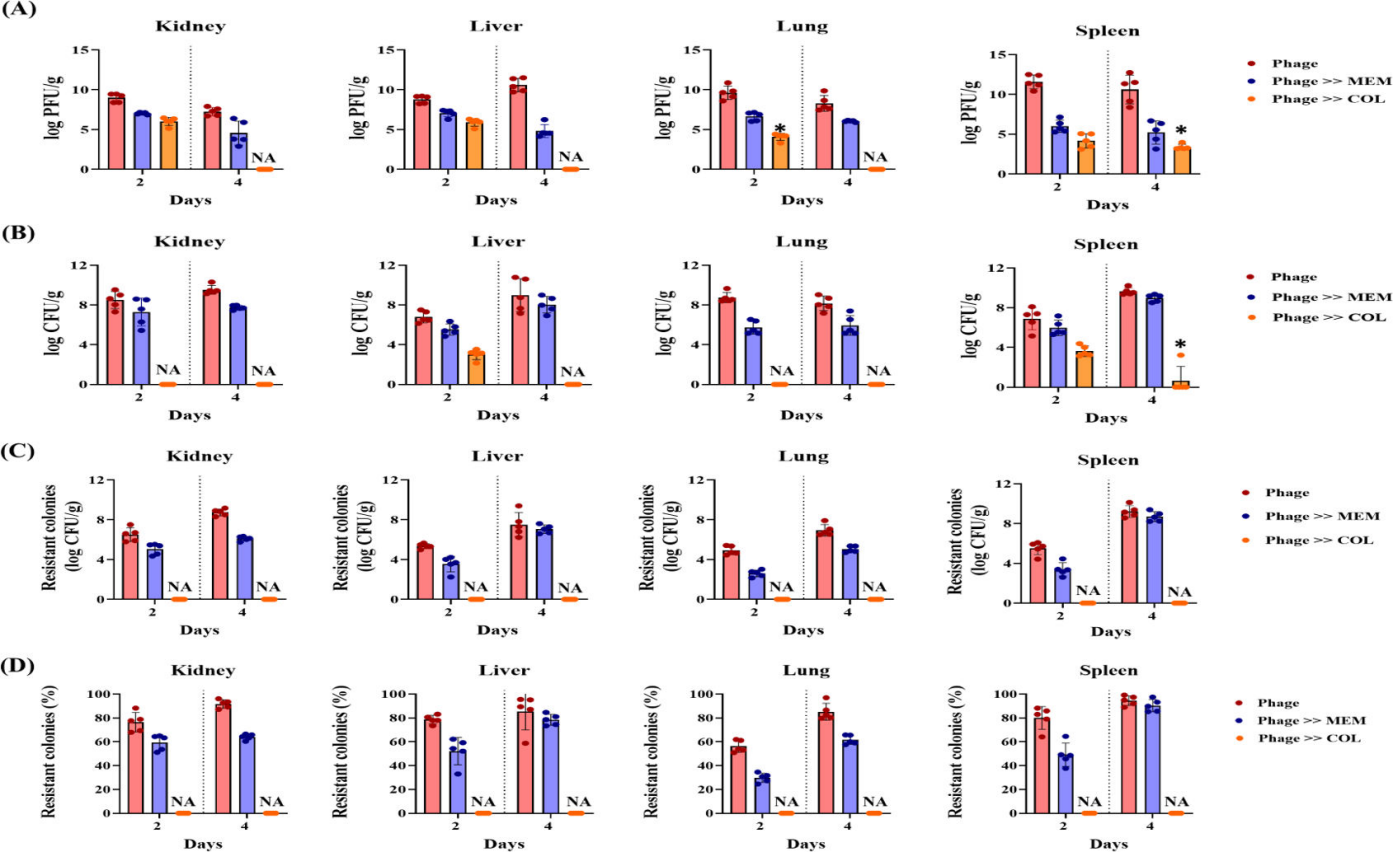
Quantification of bacterial burden, phage titers, and resistance in neutropenic mice infected with *A. baumannii* KBN10P02782 and treated with phage vB_AbaSt_W16 in combination with meropenem (MEM) or colistin (COL). Mice received either phage alone or sequential phage-antibiotic therapy (Phage → MEM or Phage → COL). Organs (kidneys, liver, lungs, spleen) were harvested on days 2 and 4 post-treatment. Treatment groups are color-coded as follows: Phage alone (red), Phage → MEM (blue), Phage → COL (orange). (****A****) Phage titers (log PFU/g). (**B**) Total bacterial burden (log CFU/g). (**C**) Resistant bacterial counts (log CFU/g). (**D**) Resistance ratio (%) calculated as (resistant CFU / total CFU) × 100. When total CFU = 0, the resistance ratio was considered not applicable (NA) and excluded from statistical analysis. Data represent mean ± SD from three independent experiments (*n* = 5 per group). Statistical analysis was performed using two-way ANOVA, followed by Bonferroni’s post-hoc test. Significant differences are indicated by asterisks (*P < 0.05, **P < 0.01).

The efficacy of sequential versus simultaneous phage-antibiotic therapy (Phage → Antibiotic vs. (Phage Antibiotic) was evaluated *in vivo*. The + survival rates following treatment with meropenem and colistin are presented in Fig. 5, while the bacterial burden, phage titers, and resistance dynamics are detailed in Fig. 6.

As shown in Fig. 5A, the survival rates at day 7 were 20% for meropenem monotherapy, 30% for simultaneous phage-meropenem treatment, and 35% for phage-meropenem sequential therapy. These values were directly obtained from the Kaplan-Meier survival analysis. However, as shown in Fig. 6, none of the meropenem-containing groups—regardless of administration timing—significantly reduced bacterial burden or suppressed the emergence of resistant colonies.

In contrast, colistin showed markedly better therapeutic effects. As shown in Fig. 5B, colistin monotherapy yielded 40% survival; simultaneous phage-colistin treatment increased survival to 60%; and sequential phage-colistin therapy (Phage → Colistin) achieved 100% survival. Bacterial burden was significantly lower in the sequential group compared to monotherapy and phage alone (Fig. 6B), and resistance levels approached zero by day 4 (Fig. 6C and D).

Importantly, statistical comparisons were performed between the sequential group and the corresponding antibiotic monotherapy group, not the SM buffer group. While meropenem did not exhibit synergy with phage vB_AbaSt_W16, sequential phage-colistin therapy significantly outperformed monotherapy, enhancing survival and suppressing resistance in CRAB infections.

## DISCUSSION

This study assessed the efficacy of phage vB_AbaSt_W16 in CRAB infections and examined whether sequential phage-antibiotic therapy improves treatment outcomes and suppresses resistance. High-dose phage therapy (MOI 10) temporarily reduced bacterial burden but promoted rapid regrowth due to resistance emergence. In contrast, sequential therapy with antibiotic administration 6 h post-phage treatment significantly improved therapeutic outcomes and effectively suppressed resistance development.

Phage therapy has been widely investigated as an alternative for treating multidrug-resistant bacterial infections, yet the rapid emergence of phage-resistant mutants remains a major limitation (14, 16). Consistent with previous findings, this study showed that MOI 0.1–1 achieved 100% survival, whereas MOI 10 resulted in only 40% survival, with a substantial increase in resistant bacterial populations. This suggests that high-dose phage therapy may exert strong selective pressure, accelerating the emergence of resistance (17, 23, 24).

Mechanisms of bacterial resistance to phages include receptor modification, CRISPR-Cas activation, and restriction-modification systems, all of which can limit the long-term efficacy of phage monotherapy (12, 13). In this study, resistant bacterial populations began to emerge from day 2 in the MOI 10 group, reaching over 90% by day 4 (Fig. 2 and 3). Notably, at MOI 0.1 on day 4, resistant colonies were not detected in the spleen, although bacterial CFU levels remained high. This may be attributed to the spleen’s role as a physiological filter, where phage-sensitive bacteria may be retained without complete clearance. In our experiments, some phage-resistant colonies appeared to regain susceptibility when re-exposed to the same phage. This observation suggests that resistance may have been mediated by transient adaptive mechanisms rather than stable genetic mutations, highlighting the potential benefit of combination therapies to overcome phage resistance.

*In vitro* experiments in this study were primarily designed to assess bacterial growth inhibition rather than to replicate therapeutic conditions. Nevertheless, the inhibitory trends observed were consistent with *in vivo* outcomes, supporting their translational relevance. PAS has been explored as a strategy to enhance bacterial eradication and delay resistance emergence (17, 23, 24). Previous studies have assessed PAS by combining phages with various antibiotics to evaluate their synergistic effects in reducing bacterial load and delaying resistance development. For example, studies on *Pseudomonas aeruginosa* and *Staphylococcus aureus* demonstrated that PAS could enhance bacterial clearance compared to monotherapy. These findings suggest that the effectiveness of PAS may be dependent on bacterial species, phage-host interactions, and antibiotic choice. In this study, synergy was primarily evaluated based on MIC and FICI values at 24 h, consistent with standard PAS testing protocols. Synergy was observed only in tigecycline (FICI = 0.225) (Table 1), while other combinations did not show synergistic interactions. However, this effect diminished after 24 h, limiting its potential for sustained bacterial suppression. As previously reported, the choice and timing of antibiotic administration are critical factors influencing the PAS efficacy (16, 25, 26). It should be noted that optical density measurements above 1.0 may be affected by light scattering; however, since our analysis focused on relative comparisons between treatment groups, this limitation is unlikely to have affected the overall conclusions.

While meropenem and colistin showed no significant PAS effects *in vitro*, their therapeutic outcomes varied considerably *in vivo*. As shown in Fig. 5A, phage-colistin sequential therapy maintained 100% survival throughout the 7-day observation period and exhibited excellent suppression of resistant bacterial populations. In contrast, phage-meropenem simultaneous therapy resulted in a survival rate of only 35% at day 7, with a limited effect on resistance suppression (Fig. 5A and 6). These findings suggest that colistin enhances phage infectivity and bacterial clearance by disrupting the outer membrane of gram-negative bacteria. In contrast, meropenem, which targets cell wall synthesis, may have limited synergy with phages.

Consistent with this, a decline in phage counts on day 4 in the phage-colistin sequential therapy group suggests that bacterial reduction may decrease phage replication, highlighting the need to consider bacterial-phage interactions for long-term therapeutic efficacy.

In our experiments, despite the fact that no PAS effect was observed when phage and colistin were administered simultaneously (*in vitro* and *in vivo*), sequential treatment (phage followed by colistin 6 h later) resulted in 100% survival in a mouse infection model. The reason for this phenomenon can be explained as follows: colistin is a bactericidal agent that disrupts the outer cell membrane of *A. baumannii* and increases membrane permeability. Phages multiply inside the host cell and lyse the bacteria, but the host must be alive for the phages to multiply. When both agents are present at the same time, if colistin kills bacteria quickly, there are fewer hosts for phage to multiply, which can reduce the effectiveness of phage therapy. In other words, if colistin kills the bacteria too quickly, limiting phage proliferation, the long-term effectiveness of the phage is reduced, and the synergistic effect is lost. It could also be the difference in PK/PD between colistin and phage. Colistin acts rapidly in a concentration-dependent manner, with efficacy declining over time. Phage, on the contrary, is a proliferative agent, meaning that a higher initial bacterial concentration favors proliferation. In simultaneous treatment, colistin will likely kill the bacteria quickly before phage has time to multiply sufficiently, reducing the long-term effectiveness of phage.

The observed 100% survival rate in the sequential phage-colistin treatment group can be attributed to several potential mechanisms. First, phage pre-treatment reduces the initial bacterial burden, allowing colistin to act more efficiently on the residual population (27, 28). Delaying colistin administration enables sufficient phage replication, which is essential for effective bacterial lysis. Once colistin is introduced, it can eliminate the remaining bacteria weakened by prior phage infection, resulting in complete bacterial clearance and reduced recurrence. Second, phage infection may enhance bacterial susceptibility to colistin by altering the outer membrane, as reported in previous studies (29). This suggests that phage-induced membrane modifications may potentiate colistin’s bactericidal activity during sequential administration. Finally, *in vivo* immune responses may synergize with sequential treatment. Phage-mediated bacterial lysis can activate host immunity, and subsequent colistin administration may complement this immune activity, contributing to effective infection resolution. Together, these factors likely explain the superior therapeutic outcomes observed with the phage-colistin sequential regimen.

In conclusion, in simultaneous treatment, colistin kills bacteria quickly, limiting the opportunity for phages to multiply, and the mechanisms of action of phages and colistin conflict, reducing long-term effectiveness. On the contrary, in sequential treatment, the phage first reduces the bacteria, allowing colistin to work more effectively; the phage has the potential to change the bacteria to increase colistin sensitivity; and the synergistic effect with the immune system can completely eliminate the bacteria. Therefore, in sequential treatment, the survival rate seems to be significantly increased due to the pre-conditioning effect of the phage, rather than just PAS. Further research is needed to optimize the dosing interval for phage and colistin and elucidate the mechanisms by which phages enhance antibiotic sensitivity.

This study demonstrates that phage-antibiotic sequential therapy is a promising strategy for treating CRAB infections, as evidenced by significantly improved survival rates and reduced bacterial burden and resistance emergence. Notably, phage monotherapy alone poses a high risk of resistance development, but the addition of antibiotics at an optimal time post-phage treatment can enhance long-term efficacy and prevent resistance formation. Previous studies have also suggested the potential of sequential therapy; for example, Torres-Barceló et al. (17) demonstrated that sequential antibiotic administration following phage treatment could suppress resistance emergence.

Chaudhry et al. (24) reported that phage-antibiotic sequential therapy significantly reduced bacterial burden in a *Pseudomonas aeruginosa* infection model. While previous studies have suggested the potential benefits of sequential therapy, this study provides the first experimental validation in *A. baumannii*, distinguishing it from prior research that primarily focused on other bacterial pathogens. These findings underscore the potential of phage-antibiotic sequential therapy as an effective and viable strategy for CRAB infection management, with implications for future clinical applications. This study not only highlights the importance of treatment timing but also offers translational insight for designing more effective phage-antibiotic combination therapies against multidrug-resistant gram-negative infections.

## SUPPLEMENTAL MATERIAL

Fig. S1 spectrum.00855-25-s0001.tif Extended heatmap data showing bacterial reduction at 12, 36, 60, and 72 h post-infection under phage vB_AbaSt_W16 and five antibiotics in *A. baumannii* LIS20133395 (ST552).

Fig. S2 spectrum.00855-25-s0002.tif Survival analysis of the healthy and neutropenic mice following high-dose phage vB_AbaSt_W16 (10¹⁴ PFU/mouse).

Supplemental material spectrum.00855-25-s0003.docx Supplemental figure legends.

ASM does not own the copyrights to Supplemental Material that may be linked to, or accessed through, an article. The authors have granted ASM a non-exclusive, world-wide license to publish the Supplemental Material files. Please contact the corresponding author directly for reuse.
