# Supplementary figures and images for: Overcoming phage resistance: efficacy of sequential phage-colistin therapy against carbapenem-resistant Acinetobacter baumannii
Source: Microbiol Spectr. 2025 Aug 14;13(10):e00855-25. doi: 10.1128/spectrum.00855-25 (PMC12502580; doi:10.1128/spectrum.00855-25)

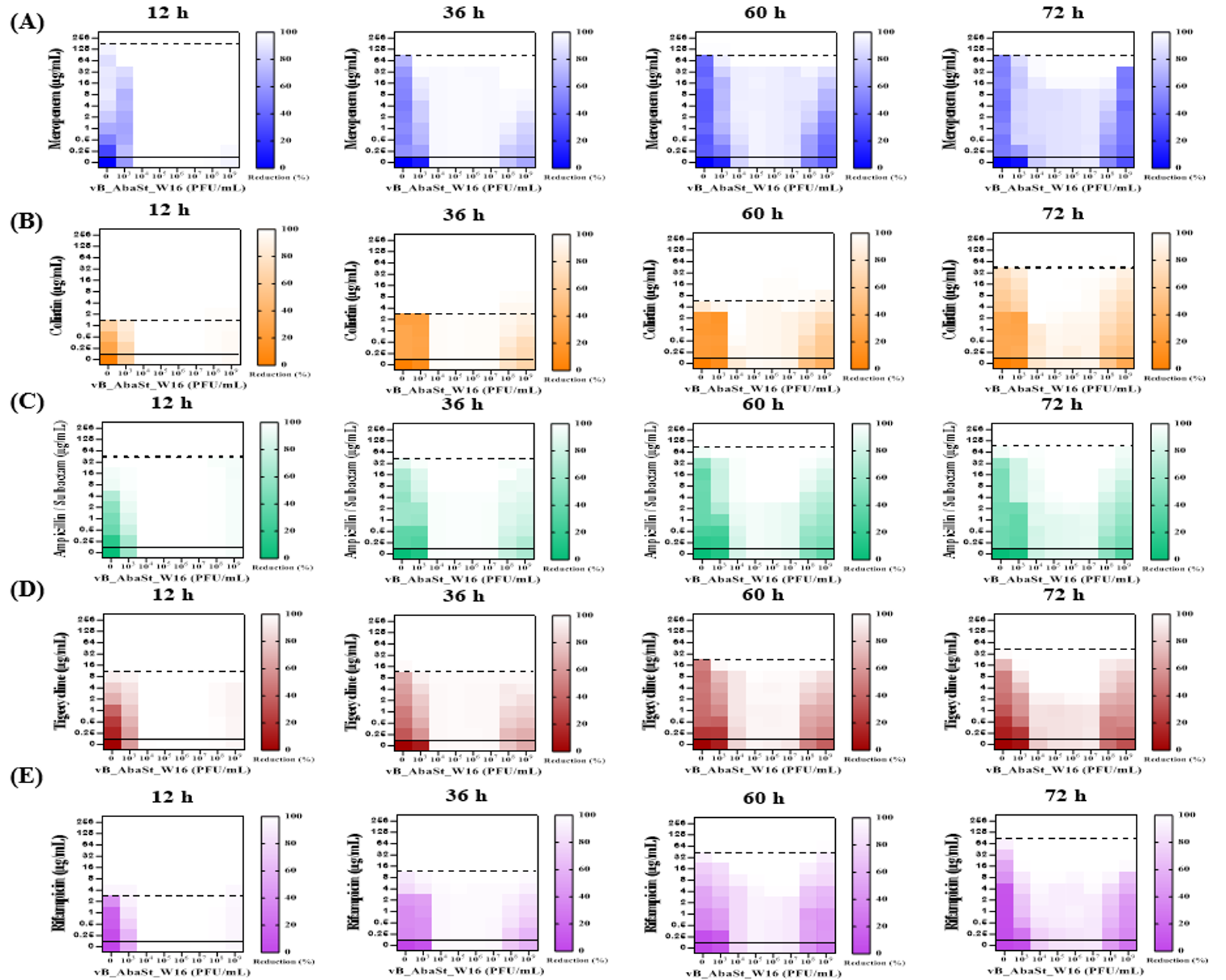

Supplement: Fig. S1 — Extended heatmap data showing bacterial reduction at 12, 36, 60, and 72 h post-infection under phage vB_AbaSt_W16 and five antibiotics in A. baumannii LIS20133395 (ST552). [file spectrum.00855-25-s0001.tif]

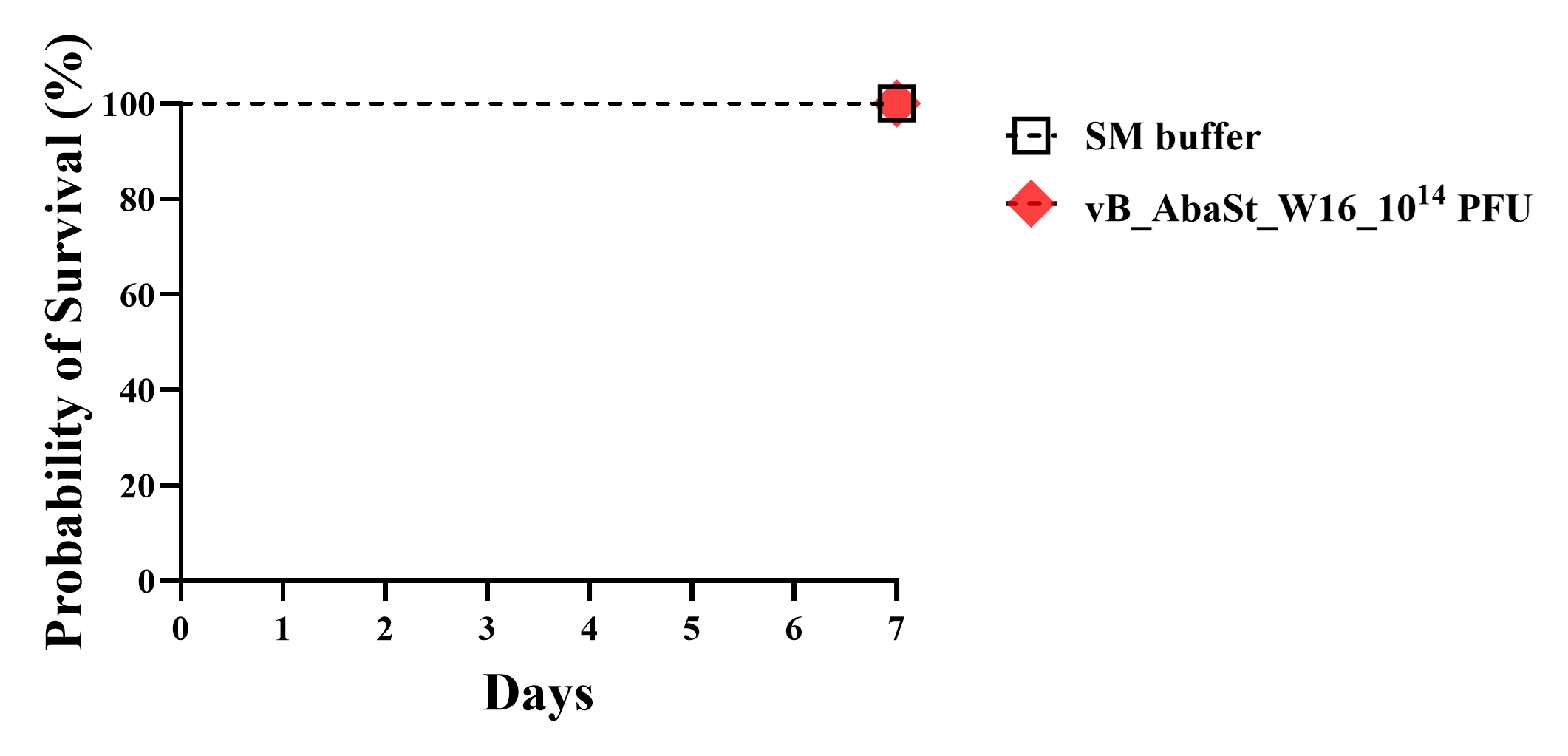

Supplement: Fig. S2 — Survival analysis of the healthy and neutropenic mice following high-dose phage vB_AbaSt_W16 (10¹⁴ PFU/mouse). [file spectrum.00855-25-s0002.tif]
