# Supplementary material for: Overcoming phage resistance: efficacy of sequential phage-colistin therapy against carbapenem-resistant Acinetobacter baumannii
Source: Microbiol Spectr. 2025 Aug 14;13(10):e00855-25. doi: 10.1128/spectrum.00855-25 (PMC12502580; doi:10.1128/spectrum.00855-25)
Supplement: Supplemental material — Supplemental figure legends. [file spectrum.00855-25-s0003.docx]

**Supplementary Figures**

**SUPPLEMENTARY FIG S1. Survival analysis of healthy and neutropenic mice following high-dose phage vB_AbaSt_W16 (10¹⁴ PFU/mouse).** Mice were treated intraperitoneally and observed for 7 days. No clinical signs or mortality were observed in any group (n = 5 per group).

**SUPPLEMENTARY FIG S2. Extended heatmap data showing bacterial reduction at 12, 36, 60, and 72 h post-infection under phage vB_AbaSt_W16 and five antibiotics in A. baumannii LIS20133395 (ST552).** (A) Meropenem, (B) Colistin, (C) Ampicillin/Sulbactam, (D) Tigecycline, (E) Rifampicin. **The data format is consistent with Figure 4.**
